# Developing Benthic Class Specific, Chlorophyll-a Retrieving Algorithms for Optically-Shallow Water Using SeaWiFS

**DOI:** 10.3390/s16101749

**Published:** 2016-10-20

**Authors:** Tara Blakey, Assefa Melesse, Michael C. Sukop, Georgio Tachiev, Dean Whitman, Fernando Miralles-Wilhelm

**Affiliations:** 1 Department of Earth and Environment, Florida International University, Miami, FL 33199, USA; tblak006@fiu.edu (T.B.); sukopm@fiu.edu (M.C.S.); whitmand@fiu.edu(D.W.); 2 GIT Consulting, Coral Gables, FL 33134, USA; georgio.tachiev@gmail.com; 3 Earth System Science Interdisciplinary Center, University of Maryland, College Park, MD 20742, USA; fwilhelm@umd.edu

**Keywords:** *chl-a*, water quality, eutrophication, optically shallow, bottom reflectance, SeaWiFS, ocean color remote sensing, validation, modeling, algorithms

## Abstract

This study evaluated the ability to improve Sea-Viewing Wide Field-of-View Sensor (SeaWiFS) *chl-a* retrieval from optically shallow coastal waters by applying algorithms specific to the pixels’ benthic class. The form of the Ocean Color (OC) algorithm was assumed for this study. The operational atmospheric correction producing Level 2 SeaWiFS data was retained since the focus of this study was on establishing the benefit from the alternative specification of the bio-optical algorithm. Benthic class was determined through satellite image-based classification methods. Accuracy of the *chl-a* algorithms evaluated was determined through comparison with coincident in situ measurements of *chl-a*. The regionally-tuned models that were allowed to vary by benthic class produced more accurate estimates of *chl-a* than the single, unified regionally-tuned model. Mean absolute percent difference was approximately 70% for the regionally-tuned, benthic class-specific algorithms. Evaluation of the residuals indicated the potential for further improvement to *chl-a* estimation through finer characterization of benthic environments. Atmospheric correction procedures specialized to coastal environments were recognized as areas for future improvement as these procedures would improve both classification and algorithm tuning.

## 1. Introduction

As an index for phytoplankton, remotely-sensed chlorophyll a (*chl-a*) has been recognized as useful for establishing a baseline for water quality conditions and for assessing current status, even in optically-complex nearshore [1,2] and inland [3] environments. The Sea-Viewing Wide Field-of-View Sensor (SeaWiFS) sensor (1997–2010) provides a valuable archive of synoptic data with one day revisit and bands tuned for retrieval of *chl-a*. However, the Ocean Color (OC) Chlorophyll algorithm, the *chl-a* algorithm currently operational for SeaWiFS, is known to overestimate *chl-a* in nearshore waters [4,5]. The OC algorithm uses empirical correlations derived from global in situ data and, therefore, cannot account for systematic differences in the bio-optical relationship that may temporarily or permanently exist in certain geographic zones [6].

Compared to satellite-derived *chl-a* for oceanic waters, nearshore environments pose challenges from colored dissolved organic matter (CDOM), suspended sediments, bottom reflectance and atmospheric conditions which, like *chl-a*, absorb blue light preferentially. Semi-analytical approaches for distinguishing between the optically significant constituents in the water column exist; however, these algorithms require accurate remote sensing reflectance (R_rs_) and may fail in the presence of negative R_rs_ so that they sacrifice coverage in comparison to empirical algorithms [6].

Few, if any, studies have evaluated improvements to the accuracy of SeaWiFS *chl-a* retrievals in optically shallow water where bottom reflectance is substantial. Le et al. [2] developed a Red-Green-Chlorophyll-Index for SeaWiFS retrieval from estuarine waters achieving uncertainties comparable to those from the OC algorithm in open ocean waters, but pixels contaminated by bottom reflectance were excluded. Using R_rs_ falling outside the transparency window (i.e., R_rs_412 and R_rs_670), Cannizzaro et al. [7] improved *chl-a* algorithm accuracy for optically-shallow water with substantial bottom reflectance. Although the algorithm developed by Cannizzaro used wavelengths available from SeaWiFS, that work utilized shipboard and mooring-collected reflectance data and did not explicitly test the applicability to satellite-based ocean color data.

The present study evaluated the ability to improve satellite *chl-a* retrieval from optically shallow coastal waters by employing algorithms specific to the pixels’ benthic class. Because of the global use of the OC algorithm, and because band-ratio algorithms have been shown to have the potential to derive *chl-a* in estuarine waters [8], the formulation of the OC algorithm was assumed for this study. The operational atmospheric correction producing Level 2 SeaWiFS data was retained because the focus of this study was on establishing the benefit from the class-specific tuning of the algorithm. The regionally-tuned models that were allowed to vary by benthic class produced more accurate estimates of *chl-a* than the single regionally-tuned model. Evaluation of the residuals indicated the potential for further improvement to *chl-a* estimation through better characterization of benthic environments.

## 2. Materials and Methods 

### 2.1. In Situ Data

**Figure 1:**
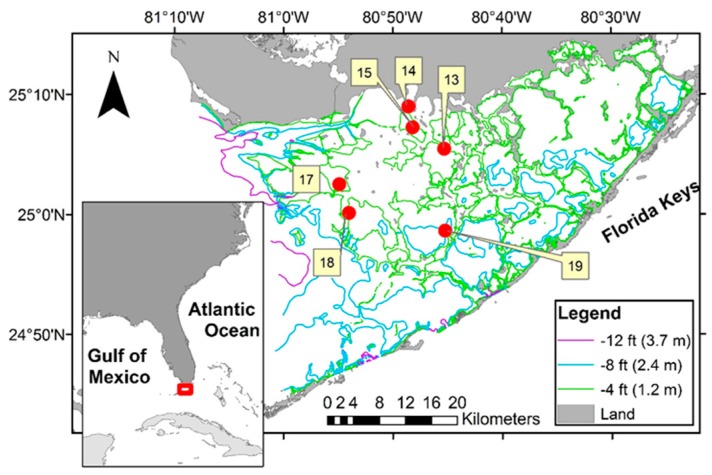
Location of study area sample stations in Florida Bay, FL, USA showing bathymetry contours as colored lines. The contours were created by the Florida Fish and Wildlife Commission based on trackline data collected in 1990 [12].

A network of water quality monitoring stations, the South Florida Water Quality Management Network, was established in Florida Bay by the Southeast Environmental Research Center at Florida International University. Field measurement of *chl-a* was conducted at water quality stations distributed across Florida Bay with data from six stations included in this study (Figure 1). Water column measurements and samples were collected every other month from July 1989 to December 1990 and monthly from March 1991 to September 2008 [9]. The data are available from the Center’s website [10]. Details on sampling methodology and laboratory analysis for *chl-a* are described by Boyer and Fourqurean [11] and Briceño and Boyer [9].

### 2.2. Satellite Data

SeaWiFS Level 2 data spanning 10 years from 1998 to 2008, as listed in Table 1, were downloaded from the NASA Goddard Space Flight Center’s website [13]. Single pixels containing in situ samples on the same day as the satellite overpass were considered as matchups to the in situ measurement. Pixels flagged for land, cloud, stray light, sun glint, high top-of-atmosphere, low R_rs_555 and atmospheric correction failure were excluded as in Bailey and Werdell [14]. Matchups were also screened to exclude data where the viewing and zenith angles exceed 60° and 75°, respectively, accounting for limitations on the atmospheric correction algorithms at extreme viewing and solar geometries [14].

### 2.3. Seagrass Class Data

A previous, supervised classification of the study area’s benthic habitat [15] was employed in the present study. A brief summary of the classification methodology is presented here. A maximum likelihood classifier was applied to remote sensing data, of the study area, that were recorded during the time-of-year when the benthic signal was assumed to dominate the variation in the top-of-water reflectances with negligible contribution from the water column. Five Landsat images, from each year in the period 2007–2011, along with Florida Bay Fisheries Habitat Assessment Program in situ surveys of seagrass cover, conducted in the spring of each year, were employed to train and validate the classifier. Data from 2009–2011 were used to train the classifier while data from 2007 and 2008 were used for validation. Pixels were classified as (1) medium-dense seagrass; (2) low seagrass; (3) sparse seagrass; or (4) turbid, as determined from three depth-invariant bands derived from the visible wavelength bands [15]. 

Sparse and low classes were combined in the present study so that a 2-class scheme was used to distinguish between all study area benthic habitats. The benthos were classified each year (1998–2008) from spring and early summer images when phytoplankton concentrations are generally low. The classifications produced from the spring/early summer data were assumed constant throughout the calendar year (from the January before the classified image to the December following the classified image). New maps, describing the mode of a 1 km radius around each 30 m Landsat grid cell, were created to account for SeaWiFS spatial resolution of 1.1 km at nadir.

### 2.4. Bio-Optical Algorithm

The empirical OC algorithm estimates were evaluated against the in situ measurements. The current version of the operational algorithm, OC4v6, relates *chl-a* to a log-transformed ratio (*X*) of remote sensing reflectance (R_rs_) [16]:

(1) $$\mathrm{chl-a}\;=\;10^{a0+a1*X+a2*X^{2}+a3*X^{3}+a4*X^{4}}$$

(2) $$X\;=\;\log_{10}\frac{\lambda_{b}}{\lambda_{g}}$$

For the OC4v6 algorithm, λ_b_ is the greatest of R_rs_443, R_rs_490, and R_rs_510, and λ_g_ is R_rs_555. The best fit polynomial was derived using the globally-distributed NASA Bio-optical Marine Algorithm Data set [17] with coefficients a1 = 0.3272, a2 = −2.9940, a3 = −1.2259 and a4 = −0.5683.

### 2.5. Statistical Analyses

Regionally-tuned algorithms, including models (1) that use alternative band ratios but no distinction by seagrass class and (2) based on band ratio *X* with coefficients tuned to benthic cover class, were tested for potential improvement of the *chl-a* estimates. All tested algorithms were derived through linear regression against the in situ data set conducted in SPSS version 21 (IBM, Armonk, NY, USA).

Two alternative band ratios, *X*_br_ and *X*_rg_, were evaluated as substitutes for *X* in optically-shallow nearshore water. Band ratio *X*_br_ retains the maximum of R_rs_443, R_rs_490, and R_rs_510 in the numerator and employs λ_r_, R_rs_670, as the reference wavelength while ratio *X*_rg_ avoids the blue wavelengths entirely:

(3) $$X_{\mathrm{br}}\;=\;\log_{10}\frac{\lambda_{b}}{\lambda_{r}}$$

(4) $$X_{\mathrm{rg}}\;=\;\log_{10}\frac{\lambda_{r}}{\lambda_{g}}$$

The in situ *chl-a* data set was segmented by benthic cover class in order to derive unique algorithms for (1) medium-dense seagrass and (2) sparse-low seagrass cover. The yearly satellite image-based seagrass classification products described previously were used to segment the *chl-a* dataset. Statistics used to assess the accuracy of the various algorithms included the adjusted R^2^ and the mean absolute percent difference (APD).

## 3. Results

Beyond the quadratic, statistical agreement (i.e., adjusted R^2^) between in situ and modeled *chl-a* generally did not improve with increasingly higher order polynomial formulations for any of the band ratios tested. Therefore, a quadratic formulation was adopted for all regressions.

### 3.1. Models Without Seagrass Distinction

The operational band ratio, *X*, employing the maximum of R_rs_443, R_rs_490, and R_rs_510 and R_rs_555, performed better than *X*_br_ and *X*_rg_ for the study area as shown in Table 2. The regression that used *X* produced positive coefficients for the linear and quadratic terms. The estimates of *chl-a* based on *X* are primarily controlled by the quadratic term with estimates increasing as the ratio of blue-to-green decreases, consistent with the absorption properties of *chl-a*.

**Figure 2:**
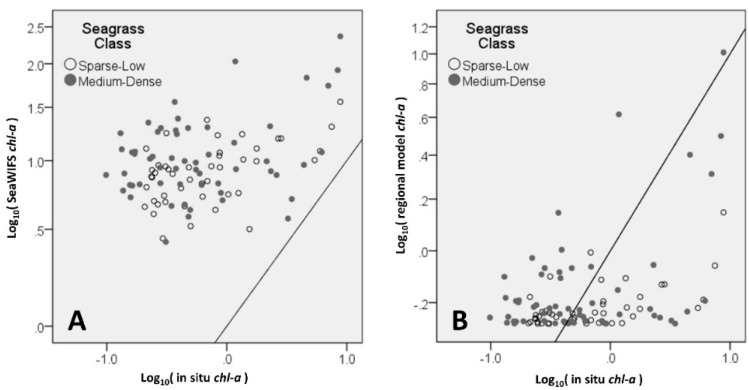
In situ measured *chl-a* versus (**A**) OC4v6 *chl-a* product and (**B**) Unified regionally-tuned model *chl-a* based on *X*.

Figure 2 plots the estimated versus the in situ $\log_{10}chl-a$ for the OC4v6 and regionally-tuned model based on *X*. From Figure 2 it is evident that the positive bias produced through the OC4v6 algorithm is less of an issue for the regionally-tuned model. 

### 3.2. Benthic Class-Specific Models

The signs and order-of-magnitude of the coefficients for the algorithms tuned to benthic class-specific data (Table 3) were the same as those for the unified, regionally-tuned model that was also based on *X*. Segmenting the data by benthic class did improve the accuracy of the resulting *chl-a* estimates in terms of the adjusted R^2^.

Table 4 shows the range of *chl-a* represented by the various algorithms tested versus the range observed in the in situ data. *Chl-a* readings over sparse seagrass were underestimated to a greater degree under the regionally-tuned model compared to the class-specific model. While the class-specific regional algorithms offer improvements over the single regional model and the OC algorithm, further improvement to predictive power may be desired before *chl-a* estimates are utilized for coastal monitoring. Therefore, assessment of the residuals by season, location and over time was conducted to investigate how further enhancement of the algorithms might be possible.

As the seagrass classification for an entire year was derived from spring or early summer data, the seasonal accuracy of the class-specific *chl-a* algorithms was of particular interest. The concern being that systematic biases in winter and fall could be representative of intra-annual changes in seagrass density that were not captured in the annual classification. The mean absolute percent differences for each season, presented in Table 5, show summer and winter retrievals to be the least accurate for the regionally-tuned, benthic class-specific models (Class-specific Overall). 

**Figure 3:**
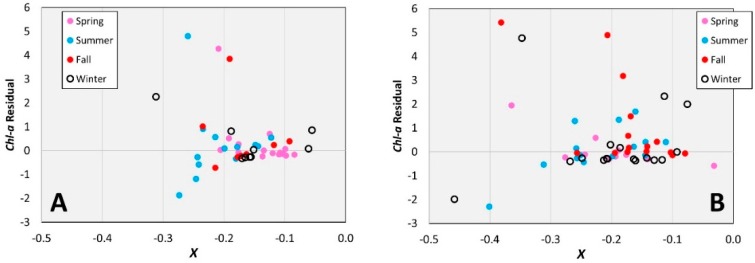
Residuals from (**A**) Sparse-low and (**B**) Medium-dense models with markers distinguished by season. The x-axis is the value of the band ratio *X* (defined in Equation (2)).

Figure 3 shows the residuals of the class-specific algorithms with markers differentiated by season. From inspection of Figure 3, the algorithm for sparse-low seagrass does not appear to produce seasonal biases. The medium-dense seagrass algorithm appears to underestimate *chl-a* somewhat in fall; however, there is no strong evidence of seasonal phenological differences in seagrass density affecting the *chl-a* estimates.

**Figure 4:**
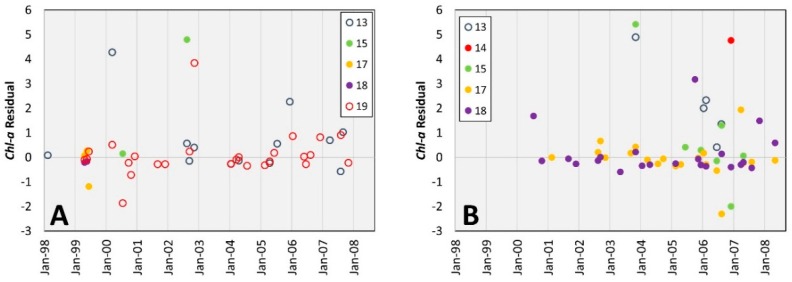
Residuals from (**A**) Sparse-low and (**B**) Medium-dense models with markers distinguished by station.

Assessment of the time-series of the residuals for the class-specific algorithms suggested biases in the medium-dense algorithm. As shown in Figure 4, estimates of *chl-a* overlying medium-dense seagrass from stations 15 and 17 are increasingly overestimated (more negative residuals) with time. As these stations were in basins associated with increasing seagrass density, the trend of increasing negative bias may be related to increased blue absorption from higher seagrass density. 

Similarly, all estimates of *chl-a* overlying medium-dense seagrass at station 13, where seagrass was typically sparse-low, were underestimated. The bias at station 13 may be related to relatively low seagrass density, and lesser blue absorption, compared to the average for the medium-dense class. These results suggest that increasing the number of seagrass classes would improve the *chl-a* estimates for the study area. By creating more divisions in the benthic classification, variations in *X* are more likely to be attributed to differences in *chl-a* as opposed to bottom reflectance.

## 4. Discussion

The benthic class-specific *chl-a* retrieval algorithms were found to greatly reduce the absolute error of retrievals compared to OC4v6, and also improve performance relative to the unified regionally-tuned model that was tested in this study. All regionally-tuned models performed significantly better than OC4v6 in capturing the dynamic range of *chl-a*, where the minimum *chl-a* estimated through OC4v6 was substantially higher than annual mean in situ *chl-a* for 10 of the 11 years of data. While the mean absolute percent difference for the benthic class-specific models remained high (77% overall) the results are encouraging given the much poorer performance of the operational algorithm in the optically-shallow conditions and the oligo- to mesotrophic nature of the study area. 

Because of the in vivo absorption peak near 676 nm, spectral bands near 676 nm have been widely used for the retrieval of *chl-a* in coastal waters [4]. The success of these algorithms is attributed to avoidance of the blue wavelengths which minimizes the impact from CDOM interference in the blue wavelengths and from atmospheric correction errors that affect blue wavelengths more strongly than green and red wavelengths [18]. While the blue to red ratio *X*_br_ was not evidenced as useful for retrieving *chl-a* concentrations in the present study, the red to green ratio *X*_rg_ achieved R^2^ values similar to those for the traditional blue to green ratio. Because *λ*_b_ and *λ*_r_ both measure reflectances associated with *chl-a* absorption, differences in phytoplankton concentration may be cancelled out in *X*_br_ (increases in phytoplankton cause increased absorption in both *λ*_b_ and *λ*_r_), accounting for the poor retrieval performance. The *X*_rg_, however, compares red absorption to a green reference and warrants further study given the known issues with satellite-retrieved blue bands. With increased attenuation from water in the red wavelengths, *X*_rg_ in optically shallow water may be influenced by variations in water column depth and variable modulation of the bottom reflectance. In this case, utilizing bands that have been corrected for variable water column depth can improve the performance of *X*_rg_ in *chl-a* retrieval.

While this study retained the functional form of the OC4v6 model, models that employ multiple band ratios may improve *chl-a* estimates where the addition of other terms could allow for finer tuning of class-specific models. Regardless of the band ratio and functional forms employed, type-specific *chl-a* retrieval algorithms present an opportunity for greater applicability of global satellite data to nearshore areas. For example, water type-specific algorithms (where water type is determined through optical classification) were shown to improve the error of multi-spectral satellite retrievals of *chl-a* over a unified model tuned for all water types without classification [19] in turbid estuaries. As benthic and water types can be identified through satellite remote sensing, type-specific algorithms offer reduction in uncertainty without sacrificing coverage.

The present study assumed negligible intra-annual variability of the seagrass class and made use of seasonally low phytoplankton to identify annual benthic class. The applicability of these assumptions to other study areas requires further study. In some study areas, the phenology of submerged aquatic vegetation may require more frequent variation of bottom reflectance although seasonal factoring may suffice in those situations. 

While better characterization of the benthos showed potential to improve *chl-a* retrieval, the capacity for this finer level of discrimination was not tested. 

The desire for finer benthic classification and the use of the blue wavelengths in the present study highlight the need for coastal atmospheric correction regimes. The atmospheric correction procedure in the nearshore is complicated by the potential for absorbing aerosols, such as smoke, dust, NO_2_ and CO_2_, and straylight contamination from nearby bright land and clouds [20]. Similar to the capability of regionally-tuned models to better represent *chl-a* in nearshore areas, atmospheric correction procedures tuned to nearshore coastal areas would result in more accurate coastal R_rs_. Reducing the satellite R_rs_ uncertainty would benefit *chl-a* retrieval directly and allow for better identification of benthic class or water types.

## 5. Conclusions

The present study demonstrates that improvements in *chl a*-retrieving algorithm performance are achievable through benthic class-specific tuning of retrieval algorithms. SeaWiFS satellite R_rs_ and satellite-derived bottom classifications were utilized for evaluation of potential reduction in the uncertainty of satellite-based *chl-a* classification. Accounting for the variation in the benthic environment by producing benthic class-specific models allowed for better representation of the water column *chl-a*. Addressing multi-spectral ocean color satellite classification uncertainty is important, as these datasets allow for the derivation of ecological baselines which can be used to detect changes in coastal system dynamics as well as be used in hindsight to assess prevailing bloom conditions and to identify the biological and physical parameters that triggered or terminated an algal event [4].

While the uncertainty in estimated *chl-a* for benthic class-specific algorithms remains substantial, type-specific models may be useful for detecting the presence of *chl-a* concentrations above a certain threshold [21] if not for estimating actual *chl-a* concentrations.

Algorithms can be optimized once benthic class and/or water types can be adequately characterized in terms of definition of classes as well as spatial and temporal variability. Therefore, the most promising opportunity to further improve satellite-based *chl-a* retrieval in nearshore areas is atmospheric correction procedures specialized to coastal environments as these procedures would improve both classification and algorithm tuning.

## Figures and Tables

**Table 1:** Summary of matchup data showing per season counts and average in situ *chl-a* annually.

Year	Spring	Summer	Fall	Winter	Total	Mean *chl-a* (mg·m^−3^)
1998				1	1	0.6
1999	6	2			8	0.5
2000	2	3	3	1	9	1.3
2001			2	3	5	0.4
2002		4	7		11	1.6
2003	1		5		6	2.7
2004	5	2	1	3	11	0.3
2005	3	3	3	7	16	1.1
2006	1	9		11	21	1.7
2007	6	4	3		13	1.1
2008	2				2	0.7
Overall	26	27	24	26	103	1.2

**Table 2:** Coefficients and goodness of fit for regionally-tuned *chl-a* retrieval models including those based on alternative band ratios.

Ratio	a0	a1	a2	Adjusted R^2^
*X*	−0.161	2.382	10.777	0.191
*X*_br_	0.003	0.646	0.394	−0.013
*X*_rg_	3.704	−3.036	0.553	0.140

**Table 3:** Coefficients and goodness of fit for benthic class-specific *chl-a* retrieval models.

Class-Specific Model	a0	a1	a2	Adjusted R^2^
Sparse-Low	−0.075	5.095	25.241	0.332
Medium-Dense	0.146	5.557	16.282	0.234

**Table 4:** Dynamic range of in situ *chl-a* compared to ranges of *chl-a* retrieved through models.

Seagrass Class	In Situ	OC4v6	Regionally-Tuned	Class-Specific
Sparse-Low	0.3–8.4	2.8–36.1	0.5–1.4	0.5–6.2
Medium-Dense	0.1–8.4	2.6–231.1	0.5–10.3	0.5–10.4

**Table 5:** Mean absolute percent difference by season for the operational SeaWiFS and the benthic-class specific model.

	Spring	Summer	Fall	Winter	All
OC4v6	1908%	2590%	1214%	2917%	2180%
Class-Specific Overall	60%	83%	54%	107%	77%
Sparse-Low	42%	80%	80%	62%	64%
Medium-Dense	85%	87%	38%	131%	87%
